# Antimicrobial resistance profiles and genome characteristics of Klebsiella isolated from the faeces of neonates in the neonatal intensive care unit

**DOI:** 10.1099/jmm.0.001862

**Published:** 2024-08-16

**Authors:** Jinghua Cui, Yanan Zhang, Xiaoran Li, Zanbo Ding, Yiming Kong, Zihui Yu, Zhaona Li, Jingjing Tong, Zunjie Liu, Jing Yuan

**Affiliations:** 1 Capital Institute of Pediatrics, Beijing 100020, PR China; 2 Beijing Obstetrics and Gynecology Hospital, Capital Medical University & Beijing Maternal and Child Health Care Hospital, Beijing 100026, PR China; 3 155th Hospital of Kaifeng, Kaifeng, Henan Province, 475003, PR China

**Keywords:** *Klebsiella* spp., multidrug-resistant, neonatal intensive care units, whole genome sequence

## Abstract

**Introduction.***Klebsiella* spp. are important bacteria that colonize the human intestine, especially in preterm infants; they can induce local and systemic disease under specific circumstances, including inflammatory bowel disease, necrotizing enterocolitis and colorectal cancer.

**Hypothesis.***Klebsiella* spp. colonized in the intestine of the neonates in the neonatal intensive care unit (NICU) may be associated with disease and antibiotic resistance, which will be hazardous to the children.

**Aim.** Our aim was to know about the prevalence, antimicrobial resistance and genome characteristics of *Klebsiella* spp. in neonate carriers.

**Methodology.** Genome sequencing and analysis, and antimicrobial susceptibility testing were mainly performed in this study.

**Results.** The isolation rates of *Klebsiella* spp. strains were 3.7% (16/436) in 2014 and 4.3% (18/420) in 2021. Cases with intestinal-colonized *Klebsiella* spp. were mainly infants with low birth weights or those with pneumonia or hyperbilirubinemia. According to the core-pan genomic analysis, 34 stains showed gene polymorphism and a sequence type (ST) of an emerging high-risk clone (ST11). Eight strains (23.5%) were found to be resistant to 2 or more antibiotics, and 46 genes/gene families along with nine plasmids were identified that conferred resistance to antibiotics. In particular, the two strains were multidrug-resistant. Strain A1256 that is related to *Klebsiella quasipneumoniae subsp. similipneumoniae* was uncommon, carrying two plasmids similar to IncFII and IncX3 that included five antibiotic resistance genes.

**Conclusion.** The prevention and control of neonatal *Klebsiella* spp. colonization in the NICU should be strengthened by paying increased attention to preventing antimicrobial resistance in neonates.

## Data Availability

The sequence data reported in this paper have been deposited in the Genome Sequence Archive (GSA) (Genomics, Proteomics & Bioinformatics 2021) at the National Genomics Data Center (Nucleic Acids Res 2022), China National Center for Bioinformation/Beijing Institute of Genomics, Chinese Academy of Sciences (GSA: CRA015414), and are publicly accessible at https://ngdc.cncb.ac.cn/gsa.

## Introduction

The intestinal microbiome plays a crucial role in the growth and development of children [1]. Enterobacteriaceae are among the first microbial colonizers of the infant intestine. The relative abundance of certain genera from this family, such as *Escherichia* and *Klebsiella*, is often increased; they can induce local and systemic disease, including inflammatory bowel disease (IBD), necrotizing enterocolitis (NEC) and colorectal cancer under specific circumstances [2,4]. *Klebsiella pneumoniae* (a representative *Klebsiella* spp.) isolated from an infant’s intestine promoted intestinal inflammation in a host lacking the immunosuppressive cytokine IL-10 [5]. Since defective IL-10 signalling has been associated with very early onset IBD [6,8], early *K. pneumoniae* colonization likely induces intestinal pathology.

Intestinal colonization in preterm infants occurs in neonatal intensive care units (NICUs). Empiric antibiotic therapy is administered to most preterm infants in the first days of life to prevent possible early-onset infections contributing to the development of a gut microbiota dominated by *Enterobacteriaceae*, *Enterococcus* and *Staphylococcus* [9,10]. Previous studies have reported that 1–10% of preterm infants harbour *Klebsiella* spp. in their faecal microbiota, and this proportion can be much higher depending on geographical location or the presence of an unstable microbiome and systemic developmental immaturity (especially concerning immune and gastrointestinal functions) [11]. *Klebsiella* spp. are one of the key microbiome members colonizing the neonate intestine, necessitating increased attention and surveillance.

Multidrug-resistant (MDR) strains of *K. pneumoniae* have increased significantly because of antibiotic abuse, resulting in rapidly emerging life-threatening nosocomial diseases in some countries. Carbapenem-resistant *K. pneumoniae* (CRK) accounted for 73.9% of 664 clinical samples in a multi-centre clinical study in China [12]. A fatal outbreak of sequence type (ST) 11 carbapenem-resistant hypervirulent *K. pneumoniae* led to increased mortality [13,14]. In Israel, faecal extended-spectrum beta-lactamase (ESBL)-carrying *K. pneumoniae* cultures were identified weekly over 4 years in all neonates in a NICU; ESBL *K. pneumoniae* acquisition decreased continuously from 94/397 (24 %) neonates in 2006 to 33/304 (11 %) in 2009 [15]. Gut-colonized *K. pneumoniae* antimicrobial susceptibility was screened in pregnant women, showing that one-third of *K. pneumoniae* isolates had acquired antimicrobial resistance genes; MDR (39.7%) and ESBL *K. pneumoniae* (14.7%) have been observed in this population [16]. *Klebsiella* spp. acquire, accumulate and transfer a myriad of antimicrobial resistance determinants, possibly representing a significant reservoir for antimicrobial resistance in the gut [17,18] and increasing the risk of resistant infections in hospital environments [19,20]. This selective pressure has likely contributed to the emergence of MDR strains in the NICU [21]. The NICU is a high-risk environment in which children are vulnerable to pathogens due to preterm birth, low birth weight, congenital malformations and immune immaturity. *Klebsiella* spp. are common pathogens found in the NICU and can cause neonatal pneumonia, sepsis, urinary tract infections and other diseases that endanger the lives of children in severe cases [22,23]. Neonates are rarely colonized by resistant bacteria on admission. Although most *Klebsiella* spp. infections are caused by endogenous intestinal carriage, and little is known about the prevalence and antibiotic resistance (AR) characteristics of *K. pneumoniae* in neonates.

In this study, *Klebsiella* spp. were isolated from faecal samples from various NICU patients in 2014 and 2021. The genotype and AR of these strains were analysed to reveal the characteristics of *Klebsiella* spp. in the intestinal microbiome of neonates in the NICU, as well as identify the possible harm that they may cause.

## Methods

### Samples and clinical information collection

We collected faecal specimens from infants admitted to the NICU department of the Beijing Obstetrics and Gynaecology Hospital, Capital Medical University, Beijing, from May to November 2014 and from March to August 2021, respectively. Rectal swabs were collected into sterile, wide-mouth collection cups and aliquoted into thirds (Cary–Blair transport media, 10% formalin for parasitology, and a vial for freezing at −20 ℃) upon enrollment. These infants suffered from pneumonia, hyperbilirubinemia, low-birth weight infants (LBWI), ABO haemolysis and so on. The studies involving human participants were reviewed and approved by the medical ethics committee of the Beijing Obstetrics and Gynaecology Hospital, Capital Medical University, Beijing, China. The number of approval documents was 2021-KY-015-1. Written informed consent to participate in this study was provided by the participants’ legal guardian/next of kin.

### Bacteria isolation and identification

Rectal swabs were then spread on primary, selective MacConkey media without antibiotics. The identities of the presumptive * K. pneumoniae* isolates were confirmed using PCR targeting the *khe* gene. *K. pneumoniae* ATCC 2146 and *Escherichia coli* ATCC 25922 were used as control strains. The experiment was performed three times.

### DNA isolation, sequencing, assembly and annotation

DNA was extracted using a TIANamp Bacteria DNA Kit (Tiangen, China) in accordance with the manufacturer’s instructions and quantified using a Quant-iT PicoGreen dsDNA Assay Kit (Invitrogen, Eugene, Oregon, US). The genomes of the strains were sequenced by Novogene Co., Ltd., Beijing, China, using the Illumina HiSeq 2500 platform. Quality trimming of 150-nucleotide paired-end reads, produced from a 500-bp genomic library, and subsequent assembly were performed using SOAP denovo v1.05. Raw data were processed in four steps, involving the removal of reads with ambiguous bases (1–90 bp), 20 bp of 100 low-quality reads (≤Q20), adapter contamination and duplicated reads. Genomic DNA was subject to whole-genome sequencing with the PacBio RS II platform on two SMRT portal 2.3.0 with over 380-fold coverage. Sequence reads were assembled with the hierarchical genome assembly process algorithm version 2 (temporary). The sequences were then screened by identity value (>50 %) and heat maps constructed with coverage value. The heat maps were prepared with the heatmap package in R (3.5.1), and cluster analysis was performed with the ward.D method. The sequence data reported in this paper have been deposited and are publicly accessible at https://ngdc.cncb.ac.cn/gsa. The accession number is GSA: CRA015414.

### Whole genome sequence (WGS) phylogenetic analysis

The core genes, new genes and pan-genome size were calculated for each combination and then extrapolated using several functions to find a best fit from the mean number at each sampling point. To better understand the evolutionary relationships and genomic variations at the gene level, the phylogenetic relationships of the *Klebsiella* spp. strains were constructed based on the complete genome sequences using mega11 software, using the maximum likelihood method with 1000 bootstrap replicates. Genomic assemblies were obtained using SPAdes v3.9.12.

### Multilocus sequence typing and serotyping of *Klebsiella*

Multilocus sequence typing (MLST) and K/O serotyping of *Klebsiella* spp. were used by Kleborate (v2.0.0) software (http://github.com/katholt/Kleborate) and the Centre for Genomic Epidemiology (http://genomicepidemiology.org/services/) through inputting and analysing the fasta format of whole genome information to obtain the assembly quality and species information.

### Antimicrobial susceptibility testing

The antibiotics including β-lactams, aminoglycosides and carbapenem commonly used in paediatric clinic treatment were selected to test antimicrobial susceptibility. The susceptibilities of *Klebsiella* spp. towards nine antibiotics, including amikacin (AMK), amoxicillin–clavulanate (AMC), ceftazidime (CAZ), cefazolin (CFZ), cefuroxime (CXM), ceftriaxone (CRO), cefoperazone (SCF), meropenem (MEM) and imipenem (IPM) were determined by the E-test method of drug-sensitive strips (Antobio, China), and the result interpretation was in accordance with Clinical and Laboratory Standards Institute guidelines. The experiment was performed three times.

### Screening of AR genes and plasmids

Sequences in all the strains were compared with those of the AR genes in the Comprehensive Antibiotic Resistance Database (CARD), which is an integrated and comprehensive online resource for curating information about AR factors. To search for the presence of specific plasmid sequences, selected plasmids were used as reference input to the blast Ring Image Generator (BRIG) together with Illumina assemblies. Except for shading (false), default values were used for all BRIG parameters. The PROVEAN platform (http://provean.jcvi.org/index.php) was used to predict alterations in the biological functions of the described proteins. A genomic map was constructed using the CGView Server.

### Statistical analysis

All data were analysed using Statistical Package for Social Sciences 20.0. Continuous variables were presented as mean ± standard deviation.

## Results

### Strain isolation and sample set characteristics

**Fig. 1.**
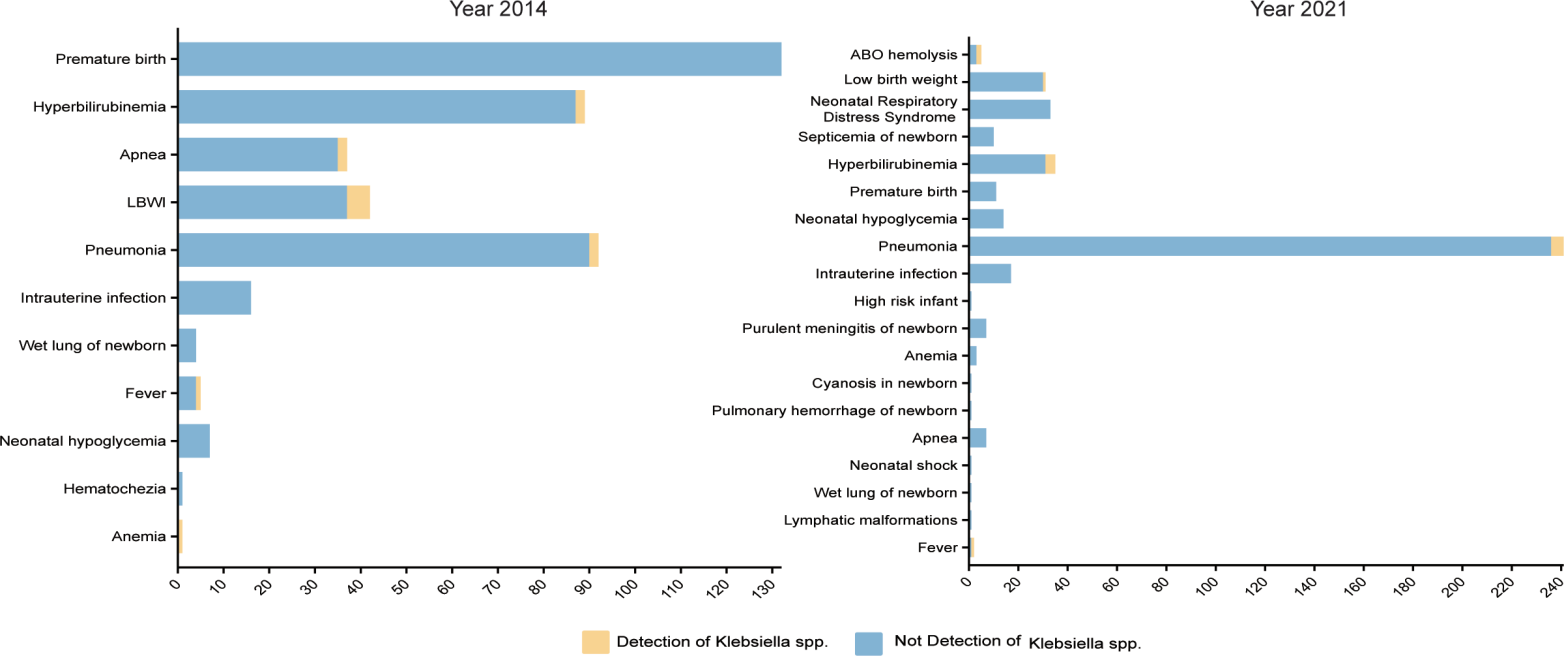
Disease distribution of neonates from the NICU in this study and the isolation rate of *Klebsiella* spp. from the intestinal tract of these neonates in 2014 and 2021.

We isolated 16 (3.7 %) *Klebsiella* spp. strains from 436 faecal samples in 2014 and 18 (4.3 %) strains from 420 faecal samples in 2021. Out of these 34 strains, 26 belonged to *K. pneumonia*, while the other eight strains were *Klebsiella michiganensis* (two strains), *Klebsiella quasipneumoniae* (two strains), *Klebsiella* (*Raoultella*) *ornithinolytica* (two strains), *Klebsiella aerogenes* (one strain) and *Klebsiella variicola* (one strain). The detailed information on these strains is shown in Table 1. In 2014, *Klebsiella* strains were isolated from the faeces of infants with low birth weights (5/42), pneumonia (2/92), hyperbilirubinemia (2/89) and apnea (2/37) (Fig. 1a). In 2021, *Klebsiella* strains were isolated from the faeces of infants with pneumonia (6/241), hyperbilirubinemia (4/26), ABO haemolysis (2/5) and low birth weights (1/31) (Fig. 1b). Cases with intestinal-colonized *Klebsiella* mainly had low birth weights, pneumonia and hyperbilirubinemia.

**Table 1.** Information on patients and isolated strains

	ID.	Age (day)	GA^1^ (month)	BW^2^ (g)	Delivery mode	Apgar score	Diagnosis	Antibiotic use	Isolated strain
1	A1042	10	36	2780	Natural labour	7,7,9	Apnea	AMC	*K. pneumoniae*
2	A1071	3	40	3220	Caesarean section	9,9,9	Anaemia	AMC	*K. pneumoniae*
3	A1078	4	37	2130	Caesarean section	10,10,10	LBWI	AMC	*K. pneumoniae*
4	A1136	5	37	2360	Caesarean section	10,10,10	LBWI	AMC	*K. pneumoniae*
5	A1147	5	39	3180	Natural labour	10,10,10	Pneumonia	AMC	*Klebsiella (Raoultella) ornithinolytica*
6	A1163	12	39	3180	Natural labour	10,10,10	Pneumonia	AMC	*K. pneumoniae*
7	A1171	4	37	2050	Caesarean section	10,10,10	LBWI	no	*K. pneumoniae*
8	A1172	7	40	3790	Natural labour	7,8,9	Apnea	PRL^3^	*K. pneumoniae*
9	A1181	9	35	2330	Caesarean section	10,10,10	LBWI	AMC	*K. pneumoniae*
10	A1185	13	39	3490	Natural labour	10,10,10	Fever	P^4^	*K. pneumoniae*
11	A1194	5	35	2100	Caesarean section	10,10,10	LBWI	AMC	*K. pneumoniae*
12	A1207	6	38	3430	Natural labour	8,10,10	Pneumonia	AMC	*Klebsiella (Raoultella) ornithinolytica*
13	A1208	6	38	3560	Natural labour	10,10,10	Pneumonia	FEP^5^	*K. pneumoniae*
14	A1214	4	38	2840	Natural labour	10,10,10	Hyperbilirubinemia	No	*K. pneumoniae*
15	A1253	2	38	3260	Forceps delivery	9,10,10	Hyperbilirubinemia	No	*K. pneumoniae*
16	A1256	12	38	3400	Caesarean section	2,3,3	Apnea	FEP	*K. quasipneumoniae* subsp. *similipneumoniae*
17	B005	10	39	3200	Caesarean section	10,10,10	Fever	no	*K. michiganensis*
18	B006	14	40	3050	Natural labour	10,10,10	Fever	CAZ	*K. pneumoniae*
19	B050	3	39	4190	Caesarean section	10,10,10	ABO haemolysis	No	*K. pneumoniae*
20	B059	6	35	2300	Natural labour	10,10,10	LBWI	No	*K. pneumoniae*
21	B131	4	40	3720	Forceps delivery	10,10,10	Hyperbilirubinemia	No	*K. pneumoniae*
22	B182	8	37	2810	Forceps delivery	10,10,10	Pneumonia	AML^6^	*K. pneumoniae*
23	B185	7	38	3030	Forceps delivery	10,10,10	Pneumonia	CAZ	*K. pneumoniae*
24	B234	3	41	3490	Caesarean section	10,10,10	Hyperbilirubinemia	No	*K. pneumoniae*
25	B260	4	39	3580	Natural labour	10,10,10	Pneumonia	AML	*K. pneumoniae*
26	B263	7	41	3770	Natural labour	10,10,10	Hyperbilirubinemia	No	*K. pneumoniae*
27	B272	14	37	2315	Caesarean section	10,10,10	Hyperbilirubinemia	No	*K. michiganensis*
28	B273	5	38	3070	Natural labour	10,10,10	ABO haemolysis	No	*K. pneumoniae*
29	B293	3	36	1920	Natural labour	10,10,10	Pneumonia	AML	*K. pneumoniae*
30	B300	6	34	2390	Natural labour	10,10,10	Pneumonia	AML	*K. quasipneumoniae* subsp. *quasipneumoniae*
31	B364	7	41	3020	Forceps delivery	10,10,10	Intrauterine infection	CAZ	*K. aerogenes*
32	B369	7	38	3490	Natural labour	10,10,10	Hyperbilirubinemia	No	*K. pneumoniae*
33	B400	14	41	4135	Caesarean section	10,10,10	Pneumonia	AML+CAZ	*K. variicola* subsp. *variicola*
34	B417	6	39	3710	Natural labour	10,10,10	Pneumonia	AML	*K. pneumoniae*

Note:1, Gestational age (GA); 2, birth weight (BW); 3, (); 4, amoxicillin-clavulanate (); 5, piperacillin (PRL);6, penicillin (P); 7, cefepime (FEP); 8, ceftazidime ();9, Aamoxycillin (AML).

### Phylogenetic diversity of whole genomes, MLST and serotypes

**Fig. 2.**
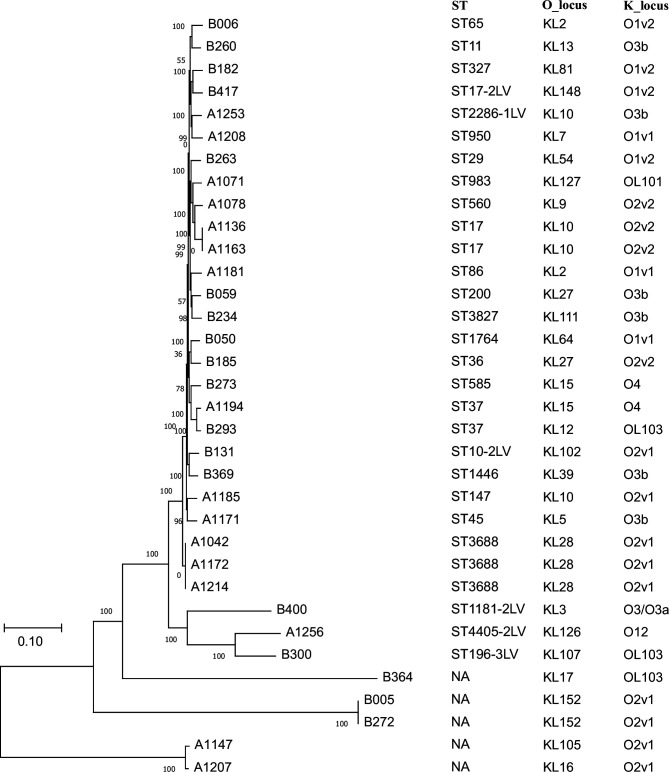
Phylogenetic diversity tree and STs and serotypes of 34 *Klebsiella* strains isolated from this study. A phylogenetic diversity tree is constructed based on the complete genome sequences using mega11 software with the maximum likelihood method with 1000 bootstrap replicates. ST and K/O serotyping are analysed by Kleborate (v2.0.0) software.

According to the core-pan genomic analysis, a tree diagram was constructed (Fig. 2). There was no cluster for the strains isolated from 2021, suggesting that no *Klebsiella* spp. transmission happened in this NICU at that time. However, strains A1136 and A1163 isolated from 2014 were in one cluster, while A1042, A1172 and A1214 were in another. Because the background of these strains was not clear, it was difficult to determine if they were from the same parent strain. In addition, the results of MLST showed that 26 *K*. *pneumoniae* strains belonging to 22 different STs were identified, indicating that these strains possessed ST polymorphism. One isolate, B260, belonged to ST11, which is the dominant ST responsible for the prevalence of CRKP worldwide and is considered an emerging high-risk clone [13]. There were 10 O serotypes and 26 K serotypes in the strains in this study. It was interesting that the same ST17 strains (A1136 and A1163) were found to possess different serotypes. All the above data are shown in Fig. 2.

### Antimicrobial susceptibility profiles of *Klebsiella* strains in this study

**Fig. 3.**
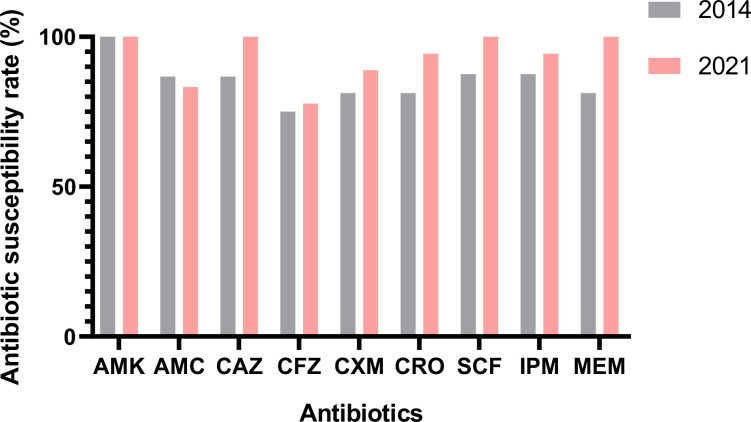
The comparison of the antibiotic susceptibility rates of the strains between 2014 and 2021.

Antimicrobial susceptibility testing revealed that the resistance rates to antibiotics were 20.6% (7/34) for CFZ, 17.6% (6/34) for AMC, 11.7% (4/34) for CXM, 11.7% (4/34) for CRO and 5.9% for CAZ, SCF, IPM and MEM, respectively. In addition, there were three strains (A1185, A1256 and B006) that were resistant to carbapenem. Eight strains (23.5%) were found to be resistant to two or more antibiotics. In particular, the two strains (A1185 and A1256) were resistant to β-lactams, aminoglycosides and carbapenems; these strains were called MDR *Klebsiella* spp. because they were not susceptible to at least three categories of antimicrobials. All the *Klebsiella* strains in this study were susceptible to AMK. All the above data are listed in Table 2. Comparing the antibiotic susceptibility rates of the strains between 2014 and 2021, it was found that the antibiotic susceptibility rates of strains isolated from 2021 were higher compared with 2014 for CAZ, CRO, SCF, IPM and MEM (Fig. 3).

**Table 2.** AR of *Klebsiella* in this study

Strain no.	Antibiotics								
	AMK	AMC	CAZ	CFZ	CXM	CRO	SCF	IPM	MEM
A1042	S	S	S	S	S	S	S	S	I
A1071	S	S	S	S	S	S	S	S	S
A1078	S	S	S	S	S	S	S	S	S
A1136	S	S	S	S	S	S	S	S	S
A1163	S	S	S	S	S	S	S	S	S
A1171	S	S	I	R	R	R	S	I	S
A1172	S	S	S	S	S	S	S	S	S
A1181	S	S	S	S	S	S	S	S	S
A1185	S	R	R	R	R	R	R	R	R
A1194	S	S	S	S	S	S	S	S	S
A1208	S	S	S	S	S	S	S	S	S
A1214	S	S	S	S	S	S	S	S	S
A1253	S	S	S	S	S	S	S	S	S
A1147	S	R	S	R	S	S	S	S	S
A1207	S	S	S	S	S	S	S	S	S
A1256	S	R	R	R	R	R	R	S	R
B005	S	S	S	S	S	S	S	S	S
B006	S	S	S	I	S	S	S	R	S
B050	S	S	S	S	S	S	S	S	S
B059	S	S	S	S	S	S	S	S	S
B131	S	S	S	S	S	S	S	S	S
B182	S	S	S	S	S	S	S	S	S
B185	S	S	S	S	S	S	S	S	S
B234	S	S	S	S	S	S	S	S	S
B260	S	S	S	S	S	S	S	S	S
B263	S	S	S	S	S	S	S	S	S
B272	S	S	S	S	S	S	S	S	S
B273	S	S	S	S	S	S	S	S	S
B293	S	R	S	R	R	R	S	S	S
B300	S	R	S	R	I	S	S	S	S
B364	S	R	S	R	S	S	S	S	S
B369	S	S	S	S	S	S	S	S	S
B400	S	S	S	S	S	S	S	S	S
B417	S	S	S	S	S	S	S	S	S
R (%)	0	17.6	5.9	20.6	11.7	11.7	5.9	5.9	5.9

Note: amikacin (), amoxicillin-clavulanate (), ceftazidime (), cefazolin (), cefuroxime (), ceftriaxone (), cefoperazone (), imipenem () and meropenem (); sensitive (S), resistant (R),, intermediary (I),; R (%), resistance percentage.

### Antimicrobial resistance genes and plasmids

Antimicrobial resistance genes and gene families were screened in all strains (Fig. S1, available in the online Supplementary Material) and identified in the eight strains found to be resistant to two or more antibiotics using the database of CARD (Table 3). Forty-six genes and gene families were identified that conferred resistance to carbapenems (*bla_NDM-1_*), β-lactams (*bla_OKP-B-2_*, *bla_SHV-67_*, *bla_SHV-81_*, *bla_TEM-1_*, *bla_DHA-1_*, *bla_OKP-A-8_*, *bla_ORN1b_*, *bla_CTX-M-3_*, *bla_CTX-M-15_* and *bla_CTX-M-55_*), aminoglycosides (*aac(3)-IId*, *aac(6')-Ib-cr*, *aadA8*, *aadA16*, *aph3-Ia*, *aph(3'')-Ib* and *aph(6)-Id*), macrolides (*mph(A)/mph(K*)), tetracyclines (*tetR*, *tetA*), ansamycins (*arr-3*), phenicols (*floR*), fluoroquinolones (*qnrB91, qnrS1* and *qnrB4*), quaternary amines (*qacH*), sulfonamides (*sul1*, *sul2* and *sul3*), bleomycin (*bleMBL*), streptomycin (*strA*) and diaminopyrimidines (*dfrA12* and *dfrA27*), along with a putative glycosyl transferase (*pmrF*). For the carbapenem-resistant strain (A1185), the prevalence of the carbapenem resistance gene *bla_NDM-1_* was screened.

**Table 3.** Antibiotic-resistant genes and plasmids screen using WGS analysis

Strain no.	Species	Antibiotics	Antibiotic-resistant genes	Plasmid code	Plasmid name
A1147	*Klebsiella (Raoultella) ornithinolytica*	AMCCFZ	bla_ORN1b_	NZ_CP0116161NZ_ AP025011.1NZ_ CP055365.1NZ_ CP067437.1	*K. oxytoca* strain CAV1335 plasmid pCAV1335-118*Raoultella ornithinolytica* strain NUITM-VR1 plasmid pNUITM-VR1_2*K. grimontii* strain RHBSTW-00165 plasmid*K. grimontii* strain KOX 60 plasmid p4
A1171	*K. pneumoniae*	CAZ(I)CFZCXMCROIPM(I)	bla_CTX-M-55_	NZ_CP082829.1NZ_CP027395.1	*E. coli* strain SCAID WND2-2021 (3/145) plasmid unnamed2*E. coli* O104:H4 strain FDAARGOS_349 plasmid unnamed1
A1185	*K. pneumoniae*	AMCCAZCFZCXMCROSCFIPMMEM	bla_CTX-M-15_bla_NDM-1_bla_SHV-67_	NZ_CP091489.1 NZ_CP029583.1	*E. cloacae* complex sp. ECL411 plasmid unnamed3*K. pneumoniae* strain DA33140 plasmid pDA33140-112
A1256	*K. quasipneumoniae*	AMCCAZCFZCXMCROSCFMEM	bla_OKP-B-2_	nd	nd
B006	*K. pneumoniae*	CFZ(I)IPM	bla_SHV-67_	nd	nd
B293	*K. pneumoniae*	AMCCFZCXMCRO	aac(6')-Ib-cr aac(3)-IId aadA16aph3-Ia aph(3'')-Ib aph(6)-Id bla_CTX-M-3_ bla_SHV-81_ bla_TEM-1_dfrA27pmrF	NZ_CP061843.1	*K. pneumoniae* strain HC139 plasmid pHC139-5copy
B300	*K. quasipneumoniae*	AMCCFZCXM(I)	aph(6)-Id bla_DHA-1_bla_OKP-A-8_strA	nd	nd
B364	*K. aerogenes*	AMCCFZ	aadA8aph(6)-IdCMY2-MIR-ACT-ECdfrA12qacH	nd	nd

In addition, we used PlasmidFinder to identify the plasmids in all the strains (Fig. S2) and found that the plasmids carried by the *Klebsiella oxytoca* strain CAV1335 plasmid *pCAV1335-118*, the *R. ornithinolytica* strain NUITM-VR1 plasmid *pNUITM-VR1_2*, the *Klebsiella grimontii* strain RHBSTW-00165 plasmid *unnamed2*, the *K. grimontii* strain KOX 60 plasmid *p4*, the * E. coli* strain SCAID WND2-2021 (3/145) plasmid *unnamed2*, the *E. coli* O104:H4 strain FDAARGOS_349 plasmid *unnamed1*, the *Enterobacter cloacae* complex sp. ECL411 plasmid *unnamed3*, the *K. pneumoniae* strain DA33140 plasmid *pDA33140-112* and the *K. pneumoniae* strain HC139 plasmid *pHC139-5copy* (Table 2). Therefore, these strains carrying the above plasmids could be resistant to one or more antimicrobial drugs at the same time.

### Complete genome sequence of *K. quasipneumoniae subsp. similipneumoniae* A1256

**Fig. 4.**
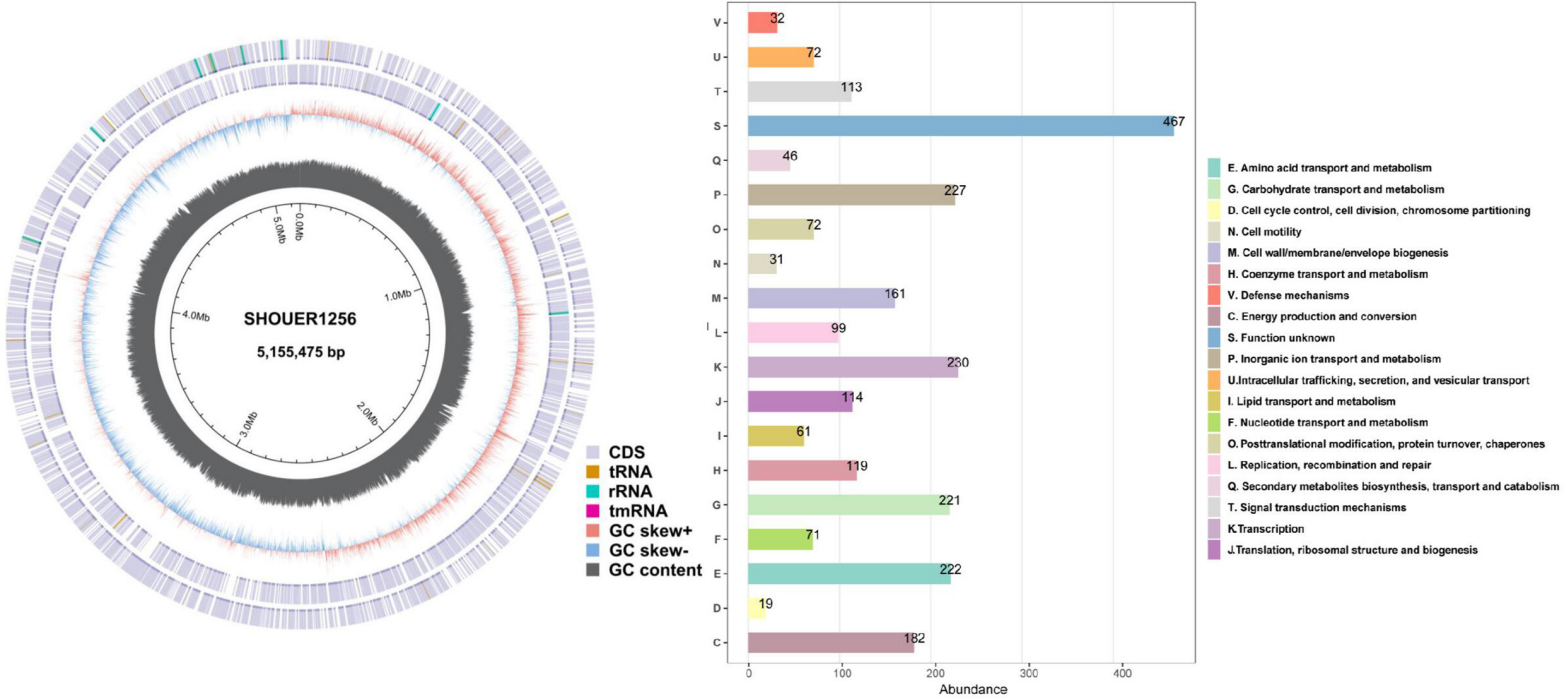
Chromosome DNA analysis of strain SHOURER1256. Circular genomic map (**a**) and COG annotation (**b**) of *K. quasipneumoniae subsp. similipneumoniae* SHOURER1256. A genomic map is constructed using the CGView Server. The database of COG is used to classify the homologous groups of proteins.

**Fig. 5.**
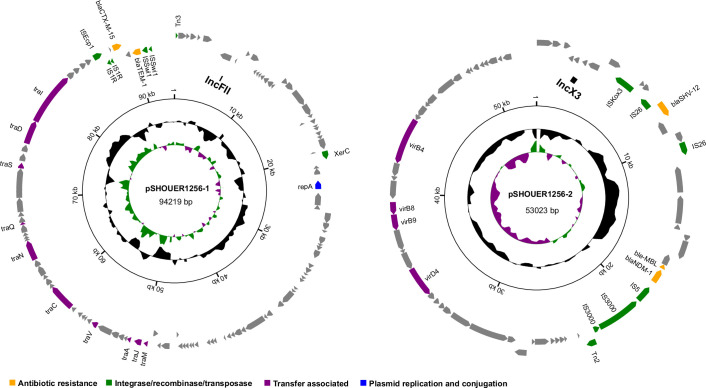
Plasmid profiles for strain SHOUER1256 isolated from this study. The plasmid map was generated using a self-developed programme. The GC content and GC Skew are represented on the distance scale (in kbp) on the inner map. The outer ring shows four different types of functional genes, including the AR gene, the integrase/recombinase/transposase gene, the transfer-associated gene and the plasmid replication and conjugation gene. The two plasmid types are IncFII and IncX3, respectively.

Strain A1256 named with SHOUER1256, was resistant to multiple antibiotics, though no antibiotic gene was identified in the draft sequence. So, we performed the complete genome sequence using Nanopore sequencing, and results showed three contigs of lengths of 5.15, 90 and 50 kb, respectively. These contigs were circular, suggesting that they were a chromosome and two plasmids similar to IncFII and IncX3 [24,25]. The guanine–cytosine (GC) content in the chromosome sequence was 58%, and the chromosome sequence encoding 4716 genes mainly include 87 tRNA, 25 rRNA and 1 transfer-messenger RNA, as shown in Fig. 4a. By comparing the genome sequence, a protein sequence was annotated into a Clusters of Orthologous Groups (COG). Each COG cluster (Fig. 4b) was composed of orthologous sequences so that the function of the sequence could be predicted. Furthermore, the two plasmids carried five AR genes (*bla_NDM-1_, bla_CTX-M-15_, bla_SHV-12_, bla_TEM-1D_* and *bleMBL*), integrase/recombinase/transposase genes, transfer-associated genes and plasmid replication and conjugation genes (Fig. 5). According to the clinical information, strain A1256 was isolated from the faeces of a male term infant 12 days after admission to the NICU after a caesarean section with apnea; the patient’s symptoms improved following the administration of intravenous FEP, and he was discharged from the hospital 20 days later. This Whole Genome Project has been deposited at GenBank under the BioProject PRJNA1082511, and SRA records was from NCBI RefSeq assemebly (GCF_037950385.1).

## Discussion

*Klebsiella* spp. sometimes contributes to systemic and life-threatening infectious diseases in neonates [26,27]. As understanding of the intestinal microbiome increases, *Klebsiella* has been revealed to be a type of symbiotic bacteria with a special role. In preterm infant faeces, microbiotas dominated by *Klebsiella* or *Enterococcus* spp. have been described as two of six preterm gut community types, with the latter type being more commonly associated with an NEC diagnosis [9]. It is important to understand the colonization of *Klebsiella* spp. in the intestines of neonates and its consequences, especially in NICU infants.

To better understand this, we selected children hospitalized in the NICU. We found that *Klebsiella* spp. were mainly isolated from infants with low birth weights (8.2%, 6/73), hyperbilirubinemia (5.6%, 6/108) and pneumonia (2.4%, 8/333), which was similar across the 2 years examined. Although the isolation rate in ABO haemolysis patients was 2/5 in 2021, the total cases were limited, making it hard to explain why this disease had a high positivity rate.

The 34 isolated *Klebsiella* strains were identified and analysed further. Although most *K. pneumoniae* infections are caused by endogenous intestinal carriage, little is known about the prevalence and microbiological characteristics of *K. pneumoniae* in newborns and the associated risk factors, including exposure to environmental sources. There is also a need to remove excess *Klebsiella* spp. from the intestine, but as these strains have high AR, they are hard to eliminate [28,29]. Gastrointestinal carriage of antibiotic-resistant *K. pneumoniae is* highly associated with severe nosocomial infections [30,31]. In this study, we also found a multi-antibiotic-resistant *K. pneumoniae* strain (A1185) that carried multiple AR genes and plasmids, including *bla_NDM-1_*, *bla_CTX-M-15_*, *bla_SHV-67_* and *pDA33140-112*. These AR genes have been reported in neonate patients infected by *K. pneumoniae* in different countries [32,34]; however, the sequence of *pDA33140-112* has not been published, although sequences were submitted to GenBank. In addition, it has been recently reported that *Klebsiella* spp. have developed resistance to antibiotics; for example, *K. quasipneumoniae* subsp. *similipneumoniae* strain ATCC 700603 is known for producing ESBLs, resulting in resistance to β-lactam antibiotics [35]. The presence of chromosomally encoded resistance genes, including *bla_KPC-2_*, *bla_TEM-1_*, *bla_OKP-B-9_*, *bla_NDM_*, *bla_CTX-M15_* and *oqxAB* supports this finding [36,37]. When isolated in the present study, this strain was found to be MDR and possessed two plasmids similar to IncFII and IncX3, carrying five AR genes (*bla_NDM-1_*, *bla_CTX-M-15_*, *bla_SHV-12_*, *bla_TEM-1D_* and *bleMBL*). Early use of antibiotics in children in the NICU can lead to changes in intestinal microbiome colonization. AR surveillance of *Klebsiella* spp., such as *K. quasipneumoniae subsp. similipneumoniae* in neonate faeces should be paid as much attention as surveillance for *K. pneumoniae*. It seems that the antibiotic susceptibility rate of the strains isolated in 2021 was increased compared to that of strains isolated in 2014, which might be correlated with the control of antibiotic use in clinics over the last few years.

Sometimes, neonates are colonized with bacteria before admission, possibly obtaining strains from their parents. For example, in the first report of meningitis in an infant due to hvKP transmitted within a family, a bacterial strain isolated from the cerebrospinal fluid of an infant could have originated from her father’s stool through familial transmission [38]. Neonates admitted to the NICU frequently receive multiple antibiotics and undergo longer hospitalization compared with normal infants. Transmission of *Klebsiella* spp. in the NICU can cause outbreaks of colonization and invasive infections among neonates [39]. Through sequencing, we tracked the homology of strains to determine whether they came from a cluster and could be transmitted further. The *Klebsiella* spp. strains isolated from the NICU in this study were found to possess polymorphism; although the same STs were found in strains, the information was not detailed enough to reveal the source of these strains. Despite this, this shows that there is a risk of transmission of *Klebsiella* spp. that are colonized in the intestinal tract of neonates.

## Conclusion

In summary, the isolation rates of *Klebsiella* strains from faecal samples from neonates in the NICU were not high in this study; however, an MDR *Klebsiella* spp. strain was found that possessed important plasmids and AR genes such as *bla_NDM-1_*. In addition, some strains had the same STs and core-pan genome types, demonstrating the risk of transmission in the NICU. To strengthen the prevention and control of neonatal *Klebsiella* spp. infection in the NICU as well as *Klebsiella* spp. colonization inducing other intestinal chronic diseases, attention should be paid to preventing faecal transmission in neonates.

## supplementary material

10.1099/jmm.0.001862 Uncited Fig. S1.

## Abbreviations

AMC: amoxicillin–clavulanate
AMK: amikacin
AR: antibiotic resistance
BRIG: Blast Ring Image Generator
CARD: Comprehensive Antibiotic Resistance Database
CAZ: ceftazidime
CMX: cefuroxime
CRK: carbapenem-resistant *K. pneumoniae*
CRO: ceftriaxone
ESBL: extended-spectrum beta-lactamase
FEP: cefepime
GC: guanine–cytosine
GSA: Genome Sequence Archive
IBD: inflammatory bowel disease
IPM: imipenem
LBWI: low-birth weight infant
MDR: multidrug-resistant
MEM: meropenem
MLST: multilocus sequence typing
NEC: necrotizing enterocolitis
NICU: neonatal intensive care unit
SCF: cefoperazone
SRA: Sequence Read Archive
ST: sequence type
WGS: whole genome sequence
