# Supplementary figures and images for: Antimicrobial resistance profiles and genome characteristics of Klebsiella isolated from the faeces of neonates in the neonatal intensive care unit
Source: J Med Microbiol. 2024 Aug 16;73(8):001862. doi: 10.1099/jmm.0.001862 (PMC11329266; doi:10.1099/jmm.0.001862)

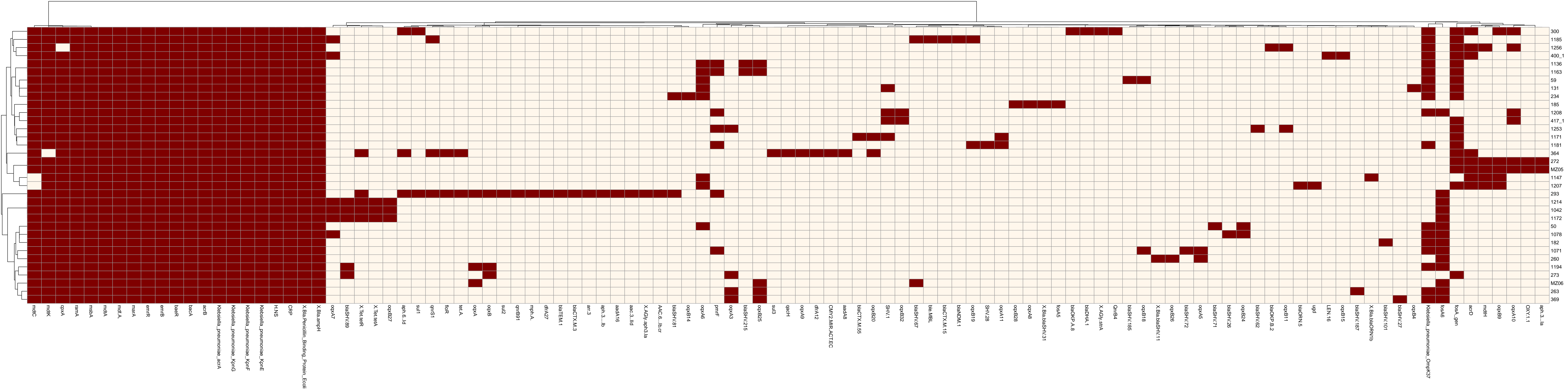

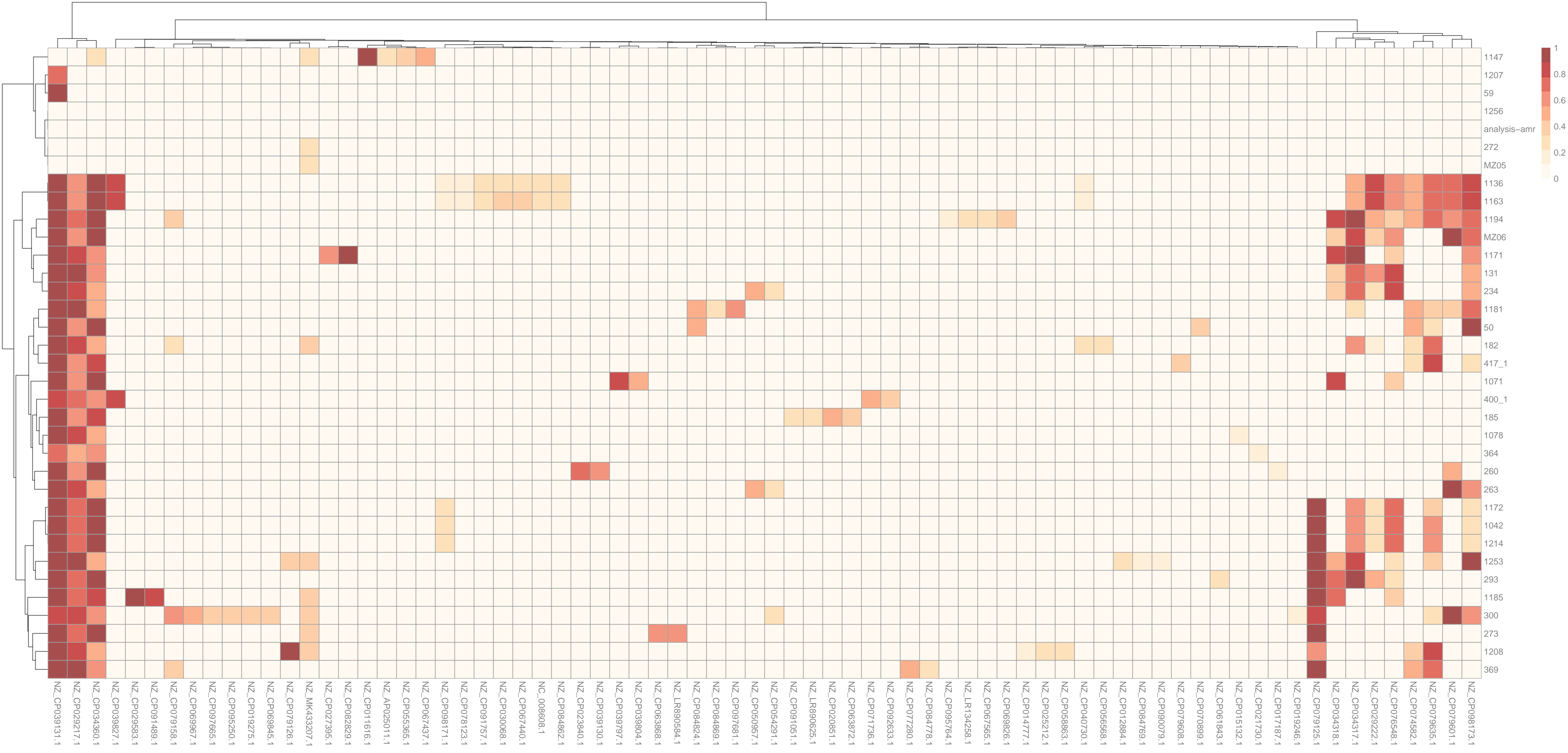

Supplement: Uncited Fig. S1. [file jmm-73-01862-s001.pdf]
